# A very rare case of an accessory subscapularis muscle and its potential clinical significance

**DOI:** 10.1007/s00276-020-02531-6

**Published:** 2020-07-12

**Authors:** Nicol Zielinska, Łukasz Olewnik, Piotr Karauda, R. Shane Tubbs, Michał Polguj

**Affiliations:** 1 Department of Anatomical Dissection and Donation, Medical University of Lodz, Lodz, Poland; 2 Department of Normal and Clinical Anatomy, Medical University of Lodz, Lodz, Poland; 3 Department of Neurosurgery, Tulane University School of Medicine, New Orleans, LA USA; 4 Department of Neurosurgery and Ochsner Neuroscience Institute, Ochsner Health System, New Orleans, LA USA; 5 Department of Anatomical Sciences, St. George’s University, Grenada, USA

**Keywords:** Subscapularis muscle, Subscapularis tendon, Accessory subscapularis muscle, Lower subscapular nerve, Rotator cuff, Quadrangular space syndrome, Compression syndrome

## Abstract

The subscapularis muscle is the largest muscle of the rotator cuff and its main function is internal rotation. It is morphologically variable in both point of origin and insertion. The presence of an accessory subscapularis muscle can lead to brachial plexus neuropathy. This report presents a very rare accessory subscapularis muscle originating from two distinct bands on the subscapularis and teres major muscles. The insertion was divided among four tendons. The fourth tendon is bifurcated. One of these was connected to the tendon of the subscapularis muscle and the other three inserted into the base of the coracoid process of the scapula. This anomalous muscle has the potential to entrap the nerves of the posterior cord such as the axillary, lower subscapular, and thoracodorsal nerves.

## Introduction

The subscapularis muscle (SM) is the largest and the most powerful muscle of the rotator cuff [10, 11]. Its origin is located on the costal surface of the scapula, which is called the subscapularis fossa. Its insertion is situated on the superior part of the humerus (in most cases, the lesser tuberosity) [11, 14, 23]. The SM limits the axillary fossa from behind. It consists of muscular and tendinous structures, both divided into several distinct parts [11]. It is innervated by the upper subscapularis (USN) and lower subscapularis (LSN) nerves [8, 9, 11]. Both the USN and LSN arise mainly from the posterior cord, which is part of the brachial plexus [8, 11]. The subscapular artery (branch of the axillary artery) supplies the muscle [11].

The function of the SM is internal rotation of the humerus, which makes it different from the other muscles of the rotator cuff (the supraspinatus, infraspinatus and teres minor muscles) [11, 16]. The SM also takes part in abduction of the shoulder. Moreover, it is a humeral head depressor and an anterior stabilizer [26].

The SM demonstrates considerable morphological variability in both origin [11] and insertion.[3, 11, 17, 27]. In rare cases, an accessory subscapularis muscle (ASM) is present [12]. Also, some SMs are fused with another muscle, for example the teres major [11].

An ASM can impose pressure on the posterior cord or nerves arising from it, leading to intense pain and problems with shoulder movement. Subscapularis tendon tears are another problem connected with this muscle. They are usually associated with glenohumeral dislocation or large multi-tendon tears of the rotator cuff. Pain located in the anterior shoulder is characteristic of subscapularis tendon tears [13].

The present report describes a very rare ASM originating as two distinct bands. One of these bands originated on the anterior surface of the SM, the other on the teres major muscle. The two bands connected with each other and created the muscle belly, which was divided among four tendons and the fourth tendon is bifurcated. Knowledge of the morphological variability of this muscle is essential for all clinicians. To our knowledge, this is the first description of such a case.

## Case report

**Fig. 1:**
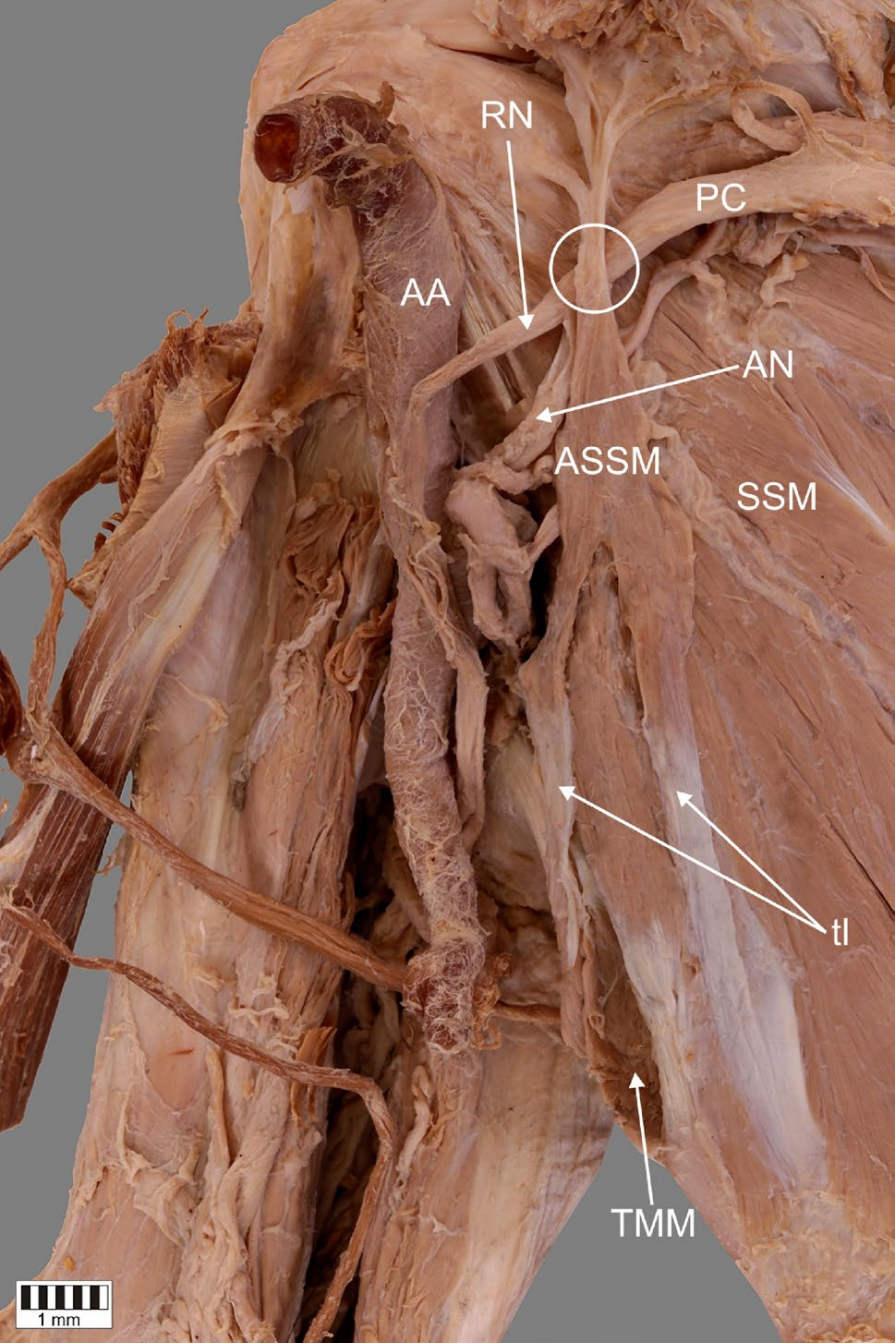
A very rare case of the accessory subscapularis muscle. Superior part of coracoid process (apex) has been removed to show bands from accessory subscapularis muscle. *SSM* subscapularis muscle, *ASSM* accessory subscapularis muscle, *PC* posterior cord of the brachial plexus, *RN* radial nerve, *AN* axillary nerve, *AA* axillary artery, *tl* tendinous band, *TMM* teres major muscle, white circle indicates a potential pressure place for posterior cord

A 66-year-old female cadaver was subjected to routine anatomical dissection for research and teaching purposes at the Department of Anatomical Dissection and Donation, Medical University of Lodz, Poland. The right upper limb was subjected to traditional anatomical dissection [1, 19–22]. During this dissection, a very rare variant of the ASM was found—Fig. 1.

The next stage of the investigation involved a detailed assessment of the ASM. The origin of this very rare structure was divided into two bands. The first band originated from an aponeurosis located on the surface of SM, 22.70 mm from the inferior angle of the scapula. It contained both tendinous and muscular parts. Its width was measured at three points: origin—7.52 mm; myotendinous junction—4.37 mm; end of the first band where the second band connected—8.19 mm. The first band was 75.93 mm long (tendinous part—58.47 mm; muscular part—17.46 mm).

The second band also consisted of two parts. The initial aponeurosis was situated on the surface of the teres major muscle. Its width was measured at three points: origin—4.75 mm; myotendinous junction—4.82 mm; end of the second band where the first band connected—3.39 mm). This band was shorter than the first (length—44.63 mm; tendinous part—27.90 mm and muscular part—16.73 mm).

The two bands joined to form one muscle belly. The length from this connection to the division into four tendons was 36.39 mm. The first tendon (length—24.51 mm) of the ASM was connected to the tendon of the SM. Its width upon passing the muscle belly was 1.79 mm and its thickness was 0.41 mm. Its width at the insertion was 2.96 mm and its thickness was 1.09 mm.

The second, third, and fourth tendons (the fourth is bifurcated) inserted into the base of the coracoid process of the scapula. The second tendon was 22.67 mm long. Its width at the myotendinous junction was 1.22 mm and its thickness there was 0.35 mm. At the insertion into the base of the coracoid process, the tendon was 2.67 mm wide and 0.65 mm thick.

The third tendon was the shortest (length—13.24 mm). Its width upon passing the muscle belly was 0.31 mm and its thickness there was 0.10 mm. Its width at the insertion was 0.39 mm and its thickness there was 0.16 mm. We can conclude that the third tendon had the lowest measurements throughout.

The fourth tendon was bifurcated (length from the origin to the division—18.06 mm). Its width at the myotendinous junction was 1.07 mm and its thickness there was 0.36 mm. The origin of first band of this tendon (4a—length after division—8.06 mm) was 0.49 mm wide and 0.08 mm thick. At the insertion into the base of the coracoid process, the band was 1.74 mm wide and 0.21 mm thick. As for the second band (4b—length after division—13.56 mm), the width of the origin was 0.92 mm and its thickness there was 0.17 mm. In turn, the insertion was 1.01 mm wide and 0.34 mm thick.

The muscle was measured with an electronic caliper (Mitutoyo Corporation, Kawasaki-shi, Kanagawa, Japan). Each measurement was repeated twice with an accuracy of up to 0.1 mm.

**Fig. 2:**
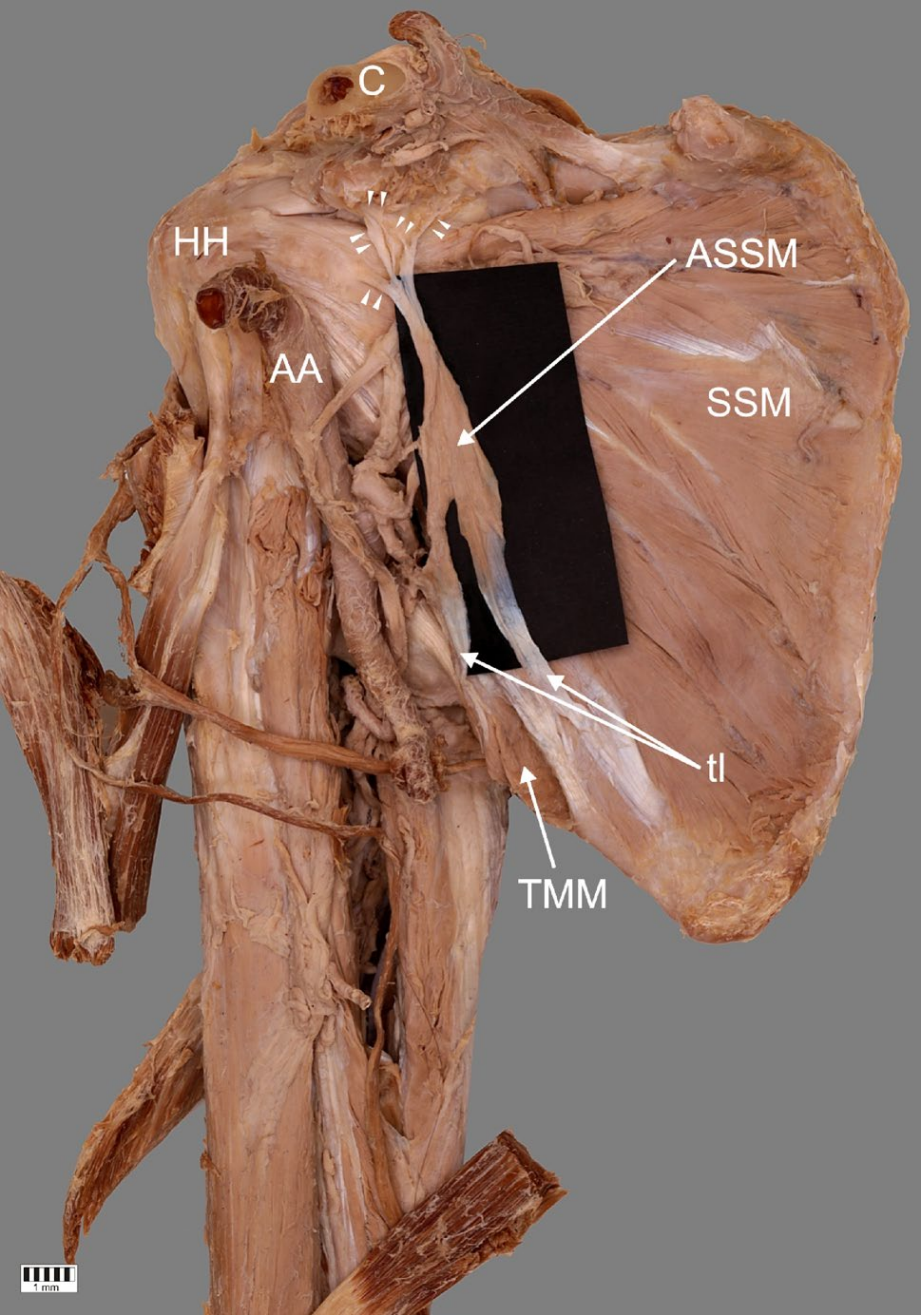
A very rare case of the accessory subscapularis muscle. Superior part of coracoid process (apex) has been removed to show bands from accessory subscapularis muscle. *ASSM* accessory subscapularis muscle, *SSM* subscapularis muscle, *tl* tendinous band, *TMM* teres major muscle, *AA* axillary artery, *HH* humeral head, *C* clavicle, white arrowheads indicate the four band insertion

It is worth mentioning that this anomalous muscle had own innervation. The lower subscapularis nerve, a branch of the posterior cord of the brachial plexus, gave off a small nerve branch to the posterior surface of the ASM. The muscle was supplied by a small arteriole arising from the subscapular artery (a large branch of the axillary artery)—Fig. 2.

During this dissection no other anatomical variations were identified.

The tables below present the collected measurements of this ASM—Table 1.

**Table 1:** Morphometric measurements of individual bands of the accessory subscapularis muscle

	Origin	
	First band	Second band
Origin	Subscapularis muscle	Teres major muscle
Length		
Tendinous part	58.47 mm	27.90 mm
Muscular part	17.46 mm	16.73 mm
Width		
Origin	7.52 mm	4.75 mm
Myotendinous junction	4.37 mm	4.82 mm
	8.19 mm	3.39 mm

	Insertion			
	First tendon	Second tendon	Third tendon	Fourth tendon
Insertion	Tendon of SM	Base of coracoid process	Base of coracoid process	Base of coracoid process
Length	24.51 mm	22.67 mm	13.24 mm	4a—18.06 + 8.06 mm 4b—18.06 + 13.56 mm
Width				
Myotendinous junction	1.79 mm	1.22 mm	0.31 mm	1.07 mm
Insertion	2.96 mm	1.67 mm	0.39 mm	4a—1.08 mm 4b—1.01 mm
Thickness				
Myotendinous junction	0.41 mm	0.35 mm	0.10 mm	0.36 mm
Insertion	1.09 mm	0.65 mm	0.16 mm	4a—0.12 mm 4b—0.34 mm

## Discussion

The ontogenesis of the SM involves fusion of three primitive muscle masses during the early stage of embryonic development. Each of these muscle masses is supplied by different nerves. There is a hypothesis that the ASM can be formed by the uppermost and the lowermost masses (separation from the parent muscles followed by fusion). After embryonic development, the lower part degenerates and is transformed into an aponeurosis [11].

Anomalies such as accessory muscle bellies have been described [18]. Although several anatomical variations in the shoulder musculature have been reported, the axillary arch muscle is the most common. This structure is also called Langer’s muscle, the pectorodorsalis muscle or the axillopectoral muscle [12]. A distinct group of anomalies of the shoulder musculature comprises variations related to the SM. The number of reported cases of this kind ranges from 0.45 to 2.6% [7, 12, 28]. They have been named the ASM, subscapularis minor muscle, marginal accessory bundle, subscapulocapsularis muscle, subscapulohumeral muscle, accessory subscapularis-teres-latissimus muscle or infraglenoidalis muscle [24].

During a dissection of 190 human cadavers, Kameda [7] noticed an additional muscle in 10 among 380 upper limbs (2.6%). They were examples of an anomalous muscle passing through the brachial plexus. Kameda [7] termed this structure an accessory subscapularis-teres-latissimus muscle and classified it into three types [7].

An ASM was described in the cadaver of a 54-year-old Japanese male by Takafuji et al. [25]. This anomalous slip was noticed in the left upper limb. In this case, the ASM had origin on the surface of the SM near the inferior angle of the scapula. It is inserted into the surface of the SM via two distinct muscle bundles [25].

An ASM was also noted by Krause et al. [12]. It had origin on the mid-region of the lateral margin of the SM. Its insertion was located on the humerus with the tendon of the SM.

Elsewhere, an ASM was found in the left axillary fossa of a 95-year-old male cadaver. This case was described by Yoshinaga. The ASM originated from the surface of the SM near the lateral border of the scapula and its insertion was fused with the capsule of the shoulder joint via a tendon [28].

The foregoing cases reveal many types of variation of the SM. This is why there are several names for anomalous structures connected to it. For example, Gruber [5] distinguished the marginal axillary bundle from the subscapularis minor muscle. Both have origin on the lateral border of the scapula in the so-called “marginal axillary groove” and insertions on the lesser tuberosity. The difference between them is related to the degree of fusion with the SM. Macalister [15] also identified two types, the subscapulocapsularis and subscapulohumeral muscles, which originate on the lateral border of the scapula. The difference between these types lies in their insertion points (subscapulocapsularis muscle—neck of the humerus and the lower part of transverse humeral ligament; subscapulohumeral muscle—lesser tuberosity and crest of the lesser tuberosity). The ASM was so called by Yoshinaga et al. [28] and Breisch [2] and its origin was located on the surface of the SM. As already mentioned, the case described by Yoshinaga had an insertion fused with the capsule of the shoulder joint via a tendon. The ASM defined by Breisch [2] inserted on the lesser tuberosity. We can also highlight the accessory subscapularis-teres-latissimus muscle, defined by Kameda [7] Its point of origin is located on the surface of the SM and its insertion point on the tendon of the SM. Manuel Staniek and Erich Brenner [24] in 2011 distinguished new type of anomalous muscle—the infraglenoid muscle—which originated on the lateral border of the scapula in the upper third of the “marginal axillary groove”. Its insertion point could be located on the crest of the lesser tuberosity, on the lesser tuberosity, or on both anatomical structures [24]. The comparison of different case reports is in Table 2.

**Table 2:** Muscle comparison among authors

Author	Name of muscle	Origin	Insertion	Additional information
Gruber [5]	Marginal axillary bundle	Lateral border of the scapula in the so-called “marginal axillary groove”	The lesser tuberosity	65%: completely or partly fused with the SM;
	Subscapularis minor muscle			20%: separated by a layer of the subscapularis facia 5%: completely independent muscle enveloped in its own fascia
Macalister [15]	Subscapulocapsularis muscle	The lateral border of the scapula	Neck of the humerus and the lower part of the transverse humeral ligament	
	Subscapulohumeral muscle		Lesser tuberosity and crest of the lesser tuberosity	
Yoshinaga et al. [28]	ASM	Surface of the SM	Fused with the capsule of the shoulder joint via a tendon	
Breisch [2]	ASM	Surface of the SM	The lesser tuberosity	
Kameda [7]	Accessory subscapularis-teres-latissimus muscle	Surface of the SM	At the tendon of SM	
Staniek, Brenner [24]	Infraglenoid muscle	The lateral border of the scapula in the upper third in the “marginal axillary groove”	The crest of the lesser tuberosity, the lesser tuberosity or both anatomical structures	

Summarizing the foregoing paragraph, our case is difficult to classify in terms of the types mentioned because there were two distinct bands of origin (one situated on the surface of the teres major muscle, which makes it unique). What is more, its insertion point is also distinctive. One of the four tendons connected to the tendon of the SM and inserted on to the lesser tuberosity, and the other three inserted into the base of the coracoid process. No structure of this kind was described in any of the studies cited above.

The present case report is the first to describe attachment to the base of the coracoid process. The insertion of the second, third, and fourth tendons of the ASM inserted into the base of the coracoid process of the scapula may have significant clinical significance. Hence, the configuration of the ASM should be considered before performing coracoid mobilization. It is possible that coracoid bone block abutment can result in the development of musculocutaneous nerve (MCN) lesions (Bristow-Latarjet). During coracoid abutment transfer by Latarjet via the deltopectoral approach, it is necessary to mobilize and retract the muscle inserting to the coracoid process, which can result in injury to the MCN: This is a known complication of anterior shoulder procedures. In addition, elongation of the MCN and changes in its angle of penetration into the muscle can result in the development of transient lesions [9]. Therefore, before procedures, MRI and CT should be performed of the area; the findings can be used to gain valuable information regarding the presence of anatomical and pathological lesions and anatomical variations of the shoulder and upper limb.

It is worth paying attention to the clinical implications of the ASM. One is the possibility of some kind of neuropathy. The ASM is separated from the primary subscapularis muscle by the posterior cord or nerves arising from it (axillary, lover subscapularis, and thoracodorsal nerves) [2, 25].

Every ASM has the potential to compress neurovascular structures. For example, the accessory muscle can cause compression of the axillary nerve, and quadrilateral space syndrome (QSS) can result [12]. The axillary nerve innervates the teres minor and deltoid muscles, which function in abduction and external rotation [6], so the main symptom of QSS is weakness and atrophy of these muscles [12]. Diffuse pain around the shoulder and paresthesia in the lateral region of the shoulder also can be caused by compression of the axillary nerve [6, 12].

The same consequence can follow pressure on the posterior cord or another nerve arising from it such as the thoracodorsal nerve, which innervates the latissimus dorsi muscle, or the lower subscapularis nerve, which innervates the teres major muscle [11]. It can lead to weakness and atrophy of these muscles.

In addition, the posterior humeral circumflex artery courses along the anterior surface of the inferior aspect of the SM and it too can be compressed by the ASM. This also leads to QSS [12]. The structure of this anomalous muscle can block flow from the axillary artery, with negative consequences. Embolization of the axillary artery or its branches is associated with subsequent cyanosis, digital ischemia, and cold intolerance [6]. Other problems associated with compression of this structure are aneurysm formation, segmental dissection, focal intimal hyperplasia, and branch vessel aneurysms [4].

In this case, we have described that the ASM was separated from the SM by the posterior cord above the division to the axillary and radial nerves, which could potentially affect compression of the posterior cord of the brachial plexus—Figs. 1, 2. In this case, symptoms characteristic of compression of every nerve arising below the point of pressure could appear. This applies to the consequences of compression of the axillary, thoracodorsal and lower subscapularis nerves, which were mentioned above. It is especially worth considering the radial nerve, which is also a branch of the posterior cord. Compression of this nerve can cause problems with straightening of the forearm, wrist, and thumb. Wrist drop also is a characteristic syndrome [11] Moreover, a sharp or burning pain and unusual sensations such as numbness and tingling can ensue [11].

We speculate that the case presented and its type of relationship to nerve structures can entail more serious effects than a situation in which only one nerve is compressed.

## Conclusions

The present case report describes a very rare ASM with an unusual structure. It can be assumed that the muscle in this case can put pressure on the posterior cord and produce several types of neuropathy. Finally, this type of accessory muscle can compress the humeral circumflex artery, leading to QSS, which is also caused by entrapment of the axillary nerve.

## Availability of data and materials

Please contact authors for data requests (Nicol Zielinska—email address: nicol.zielinska@stud.umed.lodz.pl).
